# Programming of Vascular Dysfunction by Maternal Stress: Immune System Implications

**DOI:** 10.3389/fphys.2022.787617

**Published:** 2022-03-10

**Authors:** Tiago J. Costa, Júlio Cezar De Oliveira, Fernanda Regina Giachini, Victor Vitorino Lima, Rita C. Tostes, Gisele Facholi Bomfim

**Affiliations:** ^1^Department of Pharmacology, Ribeirão Preto Medical School, University of São Paulo, Ribeirão Preto, Brazil; ^2^Health Education Research Center (NUPADS), Institute of Health Sciences, Federal University of Mato Grosso, Sinop, Brazil; ^3^Institute of Biological Sciences and Health, Federal University of Mato Grosso, Barra do Garças, Brazil

**Keywords:** maternal stress, vascular dysfunction, immune response, DOHaD (developmental origins of health and disease), fetal programming, renin–angiotensin–aldosterone system, reactive oxygen species, toll like receptor

## Abstract

A growing body of evidence highlights that several insults during pregnancy impact the vascular function and immune response of the male and female offspring. Overactivation of the immune system negatively influences cardiovascular function and contributes to cardiovascular disease. In this review, we propose that modulation of the immune system is a potential link between prenatal stress and offspring vascular dysfunction. Glucocorticoids are key mediators of stress and modulate the inflammatory response. The potential mechanisms whereby prenatal stress negatively impacts vascular function in the offspring, including poor hypothalamic–pituitary–adrenal axis regulation of inflammatory response, activation of Th17 cells, renin–angiotensin–aldosterone system hyperactivation, reactive oxygen species imbalance, generation of neoantigens and TLR4 activation, are discussed. Alterations in the immune system by maternal stress during pregnancy have broad relevance for vascular dysfunction and immune-mediated diseases, such as cardiovascular disease.

## Introduction

Fetal programming is characterized by environmental events experienced during fetal development, inducing immune and inflammatory consequences to the fetus along its lifespan (Facchi et al., 2020). There is consensus that maternal stress during pregnancy is correlated with poor offspring outcomes, and several mechanisms have been recognized to trigger intrauterine disturbances as well as fetal programming.

Maternal stress refers to the chronic or acute psychic, physical or neurological injuries that a mother experiences during any period of the pregnancy and the inability to adapt of these environmental demands (Chrousos and Gold, 1992). Several evidence has linked maternal stress contribute to the development of cardiometabolic disease in later life of offspring, including diabetes and hypertension (Reynolds et al., 2001; Perrone et al., 2016; Simeoni et al., 2018; Briffa et al., 2020). Considering that the immune response is also susceptible to *in utero* programming and that its activation directly contributes to the development of cardiovascular diseases, it is reasonable to hypothesize that the programmed *in utero* immune response may link maternal prenatal stress to vascular dysfunction in the offspring. We propose to explore original manuscripts and conceptual review with potential mechanisms that underpin maternal stress potentially modulates and programs the immune system, inducing vascular dysfunction and cardiovascular diseases in the offspring.

## Maternal Stress Exposure: Contribution of Glucocorticoids and Inflammation

During pregnancy, the maternal immune system undergoes an extensive remodeling process, allowing the fetus to adequately grow. Peter Medawar et al. demonstrated the connections between the immune system and organ transplantation, and the immunological paradox of pregnancy was uncovered: “*how does the maternal body sustain a semiallogenic fetus during pregnancy, when it would reject a semi-allogeneic graft?*”. Over time, several observations raised the concept that one of the most remarkable features from the maternal immune system is its tolerance capacity, where the mother allows the placenta-fetus unit to develop, avoiding this semi-allograft fetus rejection (Guleria and Sayegh, 2007).

Three different mechanisms were suggested to explain how maternal tolerance occurs during pregnancy (Pang et al., 2016). First, the occurrence of anatomical separation, which was ruled out since a collaborative interaction occurs between maternal decidual leukocytes and the extravillous cytotrophoblast cells from the fetus, promoting the uterine spiral arteries remodeling at early stages of pregnancy (Tilburgs et al., 2015). Second, the possibility of fetal antigens being immature, which was revoked since data from animal studies confirmed that fetal antigens are in fact mature (Billingham et al., 1954). Third, the possibility that the maternal immune system develops a reduced response, a hypothesis that was unsupported since the maternal immune system during pregnancy can respond in a highly effective manner to different pathogens. Currently, the most accepted concept is that trophoblast cells and the maternal immune system develop a cooperative network, favoring the fetal development. Over the course of the pregnancy, distinct immunological phases are observed. In fact, Mor (2008) has concluded that a successful gestation relies on how well the trophoblast will communicate with the maternal immune cells, making them work in synergy (Mor, 2008).

The first trimester of pregnancy is characterized by a remarkable pro-inflammatory environment where the maternal decidua is composed mainly by natural killer (NK) cells and macrophages, with a reduced number of dendritic cells (Bulmer et al., 1988). During this period, uterine innate immune cells display a peculiar phenotype. NK cells are not cytotoxic and are crucial for the regulation of angiogenesis and trophoblast invasion (Kopcow et al., 2005; Hanna et al., 2006). Macrophages favor trophoblast migration and the removal of apoptotic subproducts (Mor et al., 2006). In the second trimester, an anti-inflammatory profile is predominant, with a massive fetal growth and development. In the third immunological phase of pregnancy, the environment is predominantly pro-inflammatory, favoring the parturition process. The immune response readaptation during pregnancy works to prevent the semi-allogeneic fetus rejection, and participates to processes including implantation, placentation, and parturition. Different types of injuries and stress during this phase can harm the development of the fetus and cause great damage in its adult life, programming the development of several disorders and diseases.

In the developmental origins of health and diseases (DOHaD) paradigm, seminal environmental factors are known as endocrine and cardiovascular disruptors that influence the early programming of an unhealthy phenotype. Modulation in gene transcription patterns during critical stages of development such as the intrauterine or suckling phases, which are influenced by maternal exposure to stressful factors, is involved in the origin of many diseases, including obesity, type 2 diabetes, hypertension, asthma, and psychological disorders, that appear in adulthood (Burdge et al., 2007; Heijmans et al., 2008; DeVries and Vercelli, 2016; Agarwal et al., 2018; Tiffon, 2018; Wang et al., 2020).

**FIGURE 1:**
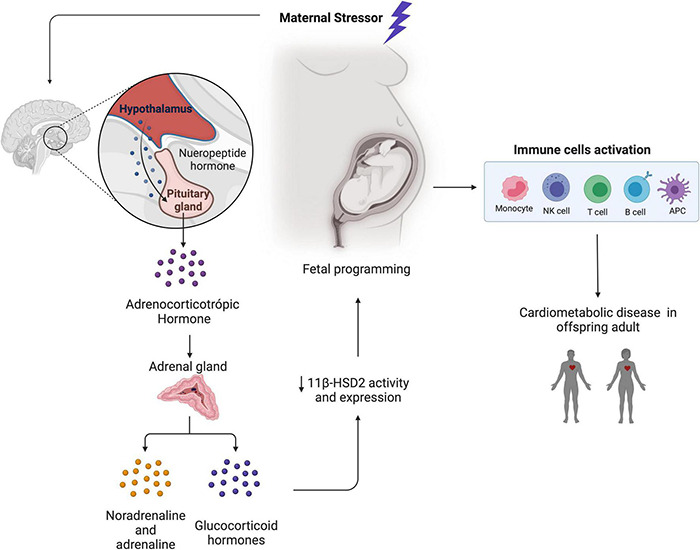
Feedback between maternal stress-induced glucocorticoid production by HPA axis (hypothalamic–pituitary–adrenal axis) activation and fetal programming induced cardiometabolic disease in offspring adult.

The food restriction during pregnancy is the most common maternal stress (Smart and Dobbing, 1971; Hales and Barker, 1992; Roseboom et al., 2001). However, obesity, diabetes (Armitage et al., 2008; Shankar et al., 2008; Agarwal et al., 2018), stressful life events, air pollution (Burris and Baccarelli, 2017; Almeida et al., 2019; Wang et al., 2020), nicotine/tobacco smoke (Butler and Goldstein, 1973; Khorram et al., 2010; Santos-Silva et al., 2013; Younes-Rapozo et al., 2013; Lisboa et al., 2017; Peixoto et al., 2018), and the use of synthetic glucocorticoids (Guo et al., 2020; Li et al., 2020; Arias et al., 2021) are important factors influencing the offspring health in later life, since they disrupt maternal and offspring physiology. Although stress induces different signaling pathways, the glucocorticoids are key mediators of stress response (Molnar et al., 2003; Moisiadis and Matthews, 2014a; Facchi et al., 2020) and beside their important role in the acute stress condition, glucocorticoids can also chronically affect the offspring brain neuronal connectivity, stability, and maturation, which can modulate hypothalamic energy controlling pathways (Arck et al., 2001; Sandman et al., 2011; Christian et al., 2013; Solano et al., 2016; Osborne et al., 2018; Schepanski et al., 2018; Nazzari et al., 2020; Figure 1).

Glucocorticoid early overexposure: a stress-induced programming-mediating factor. The role of glucocorticoids in mediating early programming of later diseases has been detailed in previous reviews (Lesage et al., 2006; Reynolds, 2013; Moisiadis and Matthews, 2014a,b; Facchi et al., 2020). Experimental and clinical studies support the hypothesis that high maternal cortisol/corticosterone levels as a consequence of stress, as well as decreased placental expression/activity of the fetal protective enzyme 11 beta-hydroxysteroid dehydrogenase type 2 (11β-HSD2), are implicated in the fetal programming of cardiometabolic diseases (Murphy et al., 1974; Bernal et al., 2017). 11β-HSD2 leads to the development of a placental barrier by oxidizing bioactive cortisol/corticosterone into bio-inactive cortisone/11-dehydrocorticosterone and thus avoiding the fetal exposure to elevated cortisol/corticosterone levels.

Maternal food restriction reduces the placental 11β-HSD2 gene expression, imprinting a smaller birthweight and reduced activity of the hypothalamic–pituitary–adrenal (HPA) axis in newborns (Lesage et al., 2001). In addition, reduction in placental 11β-HSD2 gene expression in mothers fed a low-protein diet throughout gestation is associated with smaller birthweight and hyperactive HPA axis, leading to hypertension in weaned rat offspring (Langley-Evans et al., 1996; Langley-Evans, 1997; de Oliveira et al., 2016). Pregnant women under stress conditions display lower placental 11β-HSD2 activity and lower birthweight offspring children (Stewart et al., 1995). Interesting, low activity (Stewart et al., 1995) or deleterious mutation of 11β-HSD2 (Dave-Sharma et al., 1998) was associate smaller weights in the prole. In view of this important mechanism, the deleterious effects of high concentration of glucocorticoid in immune and cardiovascular systems in the prole will be discussed in other sections.

### Epigenetic Alterations Induced by Maternal Stress

Epigenetic mechanisms, such as miRNA expression, DNA methylation, and histone modifications are prone to changes in response to stressful experiences and hostile environmental factors (Weaver et al., 2004). Altered epigenetic regulation may potentially influence fetal cardiovascular and immune response programming development across several generations (Ordovas and Smith, 2010; Babenko et al., 2012). Moreover, maternal stress during pregnancy may critically influence the density of glucocorticoid receptors in areas of the fetal brain, particularly the hippocampus, and permanently alter the sensitivity to stress throughout life (Weaver et al., 2004).

Maternal behavior produces stable alterations of DNA methylation and chromatin structure, providing a mechanism for the long-term effects of maternal care on gene expression in the offspring (Weaver et al., 2004). Epigenetic modifications were proposed as probable mechanisms of cardiovascular and metabolic programming (Babenko et al., 2012).

Epigenetic modifications occur after environmental stimuli and play a fundamental role in inflammatory gene transcription (Bayarsaihan, 2011). Indeed, integrative epigenome-wide association studies using large-scale bioinformatics analysis have reported different epigenetic marks related to several circulatory inflammation markers (Gonzalez-Jaramillo et al., 2019). Therefore, epigenetic signature alterations may exacerbate inflammatory responses and influence the risk of chronic inflammatory disease, including cardiovascular diseases (Ramos-Lopez et al., 2021).

Persistent epigenetic changes induced during early exposure to stress conditions may explain the adverse phenotypes expressed later in life (Gluckman et al., 2008). Experimental models of maternal diet during pregnancy presented DNA methylation of specific genes, which resulted in permanent phenotypic changes, such as body weight and blood pressure (Waterland and Jirtle, 2003). During the Dutch Hunger Winter between 1944 and 1945, who individuals were exposed to famine *in utero* had very different methylation patterns in genes involved in growth and metabolic disease compared with controls (Heijmans et al., 2008). These results, combined with evidence of sex-specific methylation patterns suggest that early prenatal undernutrition exposure results in change in DNA methylation and it is an important mechanism of programmed immune and cardiovascular dysregulation.

### Early Overexposure to Immune Components: A Stress-Induced Programming Event

Different prenatal stressors increase placental expression of genes involved in the immune response, including interleukin-6 (IL-6) and IL-1β, resulting in male-specific locomotor hyperactivity and increased HPA axis responses (Mueller and Bale, 2008; Bronson and Bale, 2014). A high IL-1:IL-10 ratio, which is implicated in poor coordination of glucocorticoid-immune response, has been observed in the last third trimester of pregnancy (Corwin et al., 2013). As elegantly reviewed by Schepanski et al. (2018), pregnancy stress conditions and infections expose the fetus to neurodevelopmental interferences by many cytokines, increasing the risk for neurological dysfunctions and the early programming for different health disorders in later life (Billingham et al., 1954).

Prenatal maternal stress promove alterations of cytokine profile in offspring at birth (Andersson et al., 2016). Specifically, cytokine profile of umbilical cord blood samples colected at birth from stressed mothers was associated with IL-1β, IL-6, and IL-8, cytokines that all are involved in the pro-inflammatory innate immune response, as well as IL-5 and IL4 that are prototypic cytokines of Th2 response (Wright et al., 2010; Andersson et al., 2016). It is not clear if the early exposure to cytokines high levels can influence the immune system development, but it can have some effect in the prole.

Interleukin-6, derived from the mother after pregnancy stress, effects on fetal microglia by GABAergic progenitor migration and GABAergic system development (Lussier and Stevens, 2016; Gumusoglu et al., 2017). In animal model the offspring from rats that were protein-restricted in diet during pregnancy exhibit pancreatic β-cells with increased apoptotic rate and high susceptibility to the cytokine IL-1β (Merezak et al., 2001).

Glucocorticoids are pivotal to induce physiological changes by influencing gene transcription in many tissues to maintain homeostasis, and by downregulating inflammatory processes through the cytokine-glucocorticoid negative feedback (Newton et al., 2017). By suppressing toll-like receptors (TLR) intracellular signaling, glucocorticoids inhibit the inflammatory process by different mechanisms. Glucocorticoids increase the levels of endogenous inhibitors of TLRs, such as the mitogen-activated protein kinase (MAPK) phosphatase-1 [MKP-1, also known as dual specificity phosphatase-1 (DUSP-1)] and glucocorticoid-induced leucine zipper (GILZ) and inhibit transcription factors that stimulate proinflammatory mediators, such as activator protein-1 (AP-1), nuclear factor kappa B (NF-κB), and type I interferon (IFN)-regulatory factor (IRF) (Chinenov and Rogatsky, 2007).

On the Other hand, some authors have described that the glucocorticoids induce the expression TLR4 TLR2, whose expression is commonly increased by proinflammatory cytokines such as TNF-α and IL-1β (Shuto et al., 2002; Homma et al., 2004; Chinenov and Rogatsky, 2007). In this context, increased TLR2 on epithelial cells stimulates the secretion of the cytokines such as IL-6 and IL-8. A connection between high levels of IL-6 and cardiovascular disease (Qu et al., 2014), as well as an association between IL-8 and high risk of coronary artery disease (Zhang et al., 2019a,b) have also been reported.

Despite the paradoxical crosstalk, it is important to mention that the cellular mechanism by which glucocorticoids downregulate chemokine expression and inflammatory cytokine production, may happen in cases, for example, where NF-κB addresses the systemic inflammation-associated acquired glucocorticoid resistance (Meduri et al., 2005, 2009; Newton et al., 2017). Considering the concept of DOHaD, the lack of glucocorticoids repressor effects on the inflammatory process during stress adversity in critical developmental stages of life may be considered a drawback, since it can contribute to a proinflammatory environment and negatively influence the relationship between maternal stress during pregnancy and offspring outcomes, as long-term consequences (Elenkov et al., 2005; Newton et al., 2017).

## Maternal Stress and Fetal Programming of Cardiovascular Dysfunction

Cardiovascular disease is the leading cause of death worldwide (Virani et al., 2021). The vascular system is frequently involved in the pathophysiological processes of cardiovascular diseases. Endothelial dysfunction, inflammation, and remodeling are hallmarks of vascular dysfunction in cardiometabolic diseases, such as diabetes, hypertension, dyslipidemia, and obesity (Petrie et al., 2018). Therefore, the impact, as well as the mechanisms mediating fetal programming in the vasculature have been systematically evaluated.

Vascular dysfunction is characterized by reduction of the capacity of the vessel to maintain the tonus and blood flow, increasing vascular resistance as a result of vascular hypercontractility, reduced vasodilatation, endothelial damage, and vascular remodeling. Several mechanisms may induce vascular dysfunction, including ROS, immune activation, renin–angiotensin–aldosterone system (RAAS), and the sympathetic nervous system (Touyz et al., 2018). The potential mechanisms by which maternal-stress programs vascular dysfunction in adult offspring, contributing to greater incidence of hypertension, will now be discussed.

### Food Restriction-Induced Cardiovascular Programming

Food restriction favors low birth weight in humans, correlating with high incidence of cardiovascular disease in adulthood, a feature referred to as the Barker hypothesis or the fetal origins of adult-onset disease (Hoy et al., 1999; Petrie et al., 2018).

The male offspring from pregnant rats submitted to a protocol of diet restriction during pregnancy (50% normal intake) were evaluated from the age of 4 to 14 weeks. Intrauterine food restriction favored high blood pressure levels and a reduced endothelium-dependent relaxation response, and oxidative stress was a contributor to this process (Franco Mdo et al., 2002). Nicotinamide adenine dinucleotide phosphate hydrogen (NADPH) oxidase was identified as an important source of superoxide anion (⋅O_2_^–^) generation, a process that was prevented by *in vitro* NADPH oxidase inhibition (Franco Mdo et al., 2003a), as well as *in vivo* antioxidant treatment of the offspring with ascorbic acid (vitamin C) or alpha-tocopherol (vitamin E) (Franco Mdo et al., 2003b). Prenatal antioxidant vitamin supplementation in rats exposed to a food restriction protocol during pregnancy prevented blood pressure increases and restored endothelium-dependent relaxation in the adult offspring (Franco Mdo et al., 2009). *In vitro* infusion with tetrahydrobiopterin (BH4), a cofactor for endothelial nitric oxide synthase (eNOS) activity, reverted endothelial dysfunction, favoring nitric oxide (NO) production and reduction of oxidative stress in the microvasculature (Franco Mdo et al., 2004).

Interestingly, reduced leukocyte migration was observed in offspring (8–9 weeks of age) from undernourished mothers, as a result of reduced adhesion molecule expression in leukocytes (L-selectin) and endothelial cells [P-selectin and intercellular adhesion molecule-1 (ICAM-1)] (Landgraf et al., 2005). Vascular structure may also be affected by intrauterine undernutrition, which leads to vascular smooth muscle cell hypertrophy in adult offspring (Khorram et al., 2007).

Sex-specific differences were observed in 6-month old adult offspring from mothers submitted to an undernutrition protocol during pregnancy. While males displayed augmented blood pressure levels and increased plasma metalloproteinase-9 activity, in females no differences were observed. However, females displayed smaller mesenteric resistance artery diameter, reduced cellularity of smooth muscle cells, among other structural alterations, showing that undernutrition favors later life microvasculature remodeling in both sexes, regardless of changes in blood pressure levels (Gutierrez-Arzapalo et al., 2020).

Intrauterine growth restriction (IUGR) imposed to baboons *(Papio species)*, using a protocol of 70% of food restriction during pregnancy to the end of lactation showed alterations in vascular parameters of young adult offspring, without changes in blood pressure. Augmented systolic velocity, reduced femoral and iliac artery lumen area, and reduced external iliac artery distensibility were observed in male IUGR offspring. These observations suggest that vascular remodeling observed in IUGR during fetal life somehow modulates vascular alterations later in life (Kuo et al., 2018).

### Obesity-Induced Cardiovascular Programming

Epidemiological studies demonstrated that maternal nutrient restriction or overnutrition during pregnancy predispose offspring to a much higher prevalence of obesity, glucose intolerance, insulin resistance, hypertension, vascular dysfunction, and heart disease (Rankin et al., 2010; Dong et al., 2013; Zambrano and Nathanielsz, 2013). While maternal nutrient restriction during pregnancy may trigger an increase in the tissue-specific glucocorticoid receptor, the maternal obesity induced increase in fetal hormones such as leptin and insulin, nutrients such as fatty acids, triglycerides and glucose, and inflammatory cytokines (Whorwood et al., 2001; Sullivan et al., 2011). Prenatal dietary fat exposure predisposes offspring to postnatal dietary fat-induced cardiac hypertrophy and contractile defects possibly *via* lipotoxicity, glucose intolerance, and mitochondrial dysfunction (Turdi et al., 2013). Clinical studies observed that the obesity during pregnancy induced elevation of blood pressure and cardiovascular disease in adult offspring (Gaillard, 2015).

Moreover, the maternal nutritional environment is closely associated with fetal heart and vascular development and function (Dong et al., 2008). Maternal obesity impairs fetal cardiomyocyte contractility through altered intracellular Ca^2+^ handling, overloading fetal cardiomyocyte intracellular Ca^2+^ and aberrant myofilament protein composition in sheep (Wang et al., 2019). Rats exposed *in utero* to mild maternal diet-induced obesity presented specific epigenetic modulations of matrix metalloproteinases, collagens, and potassium channels genes in association with an outward remodeling and perturbations in the endothelium-dependent vasodilation pathways in small mesenteric arteries (Payen et al., 2021).

### Hypoxia-Induced Vascular Programming

Fetal hypoxia has been linked to fetal programming of cardiovascular, renal, cerebral, and metabolic dysfunctions. Intrauterine hypoxia is frequently related to IUGR and can be induced for different maternal stress, including food restriction or obesity. It is reasonable to link oxidative stress and impairment of vascular development as mechanisms mediating intrauterine hypoxia, considering that placental dysfunction creates an ischemic environment (Fajersztajn and Veras, 2017).

Adult offspring coming from a hypoxic-environment during pregnancy associate to postnatal salt dietic display reduced endothelium-dependent relaxation. Still, larger vessels in these animals exhibit remodeling characterized by augmented collagen deposition, and reduced elastin (Walton et al., 2016). Similar observations were made in rat aortas, along with cardiac remodeling (Benny et al., 2020). IUGR also favors arterial remodeling in the adult offspring, as observed in sheep (Dodson et al., 2014).

Gestational intermittent hypoxia leads to vascular dysfunction in male offspring, due to reduced anti-contractile activity of perivascular adipose tissue, *via* epigenetic modification on the adiponectin gene promoter (Badran et al., 2019). Low oxygen levels activate the hypoxia-inducible factor 1 (HIF-1) complex, inducing epigenetic regulation, a topic that will be addressed later in this review.

### Glucocorticoid-Induced Vascular Prenatal Programming

Glucocorticoid production is important during pregnancy. In normal pregnancies, cortisol raises significantly close to the pregnancy midterm, reaching levels around 350 ng/mL (Wieczorek et al., 2019) and remaining relatively stable until labor, when cortisol raises again (Carr et al., 1981). The abundance of glucocorticoids occurs to ensure fetal organs maturation close to birth (Solano et al., 2016). Its excess is buffered in the maternal blood by the corticosteroid-binding globulin transporters, globulins that raise during pregnancy (Kumsta et al., 2007). In fact, stress-induced secretion of glucocorticoids impacts on energy stores mobilization, enhances neural function and impact on cardiovascular responses (Sapolsky et al., 2000). Yet, glucocorticoid receptors are expressed in the uterus and the target deletion of these receptors in the uterus results in infertility, inflammation and poor decidualization due to incorrect immune cell recruitment (Whirledge et al., 2015).

High doses of exogenous glucocorticoids have been used in experimental models to directly assess the role of glucocorticoids in triggering prenatal stress conditions. Therefore, intrauterine glucocorticoid exposure due to maternal stress conditions and exogenous glucocorticoid sources causes deleterious effects on vascular function in later life (Lamothe et al., 2021) and alterations of vasoactive substances may be involved.

Early gestational dexamethasone administration augments offspring blood pressure levels in sheep and favors increased contractile response to angiotensin II (Ang II), phenylephrine and thromboxane analogues (U-46619) in coronary arteries (Roghair et al., 2005). Augmented contractile response to Ang II was partially mediated by endothelial ⋅O_2_^–^ overproduction, through enhanced NADPH oxidase activity (Roghair et al., 2008). The mechanisms contributing to hypertension in a similar experimental model were related to compensatory endothelial-dependent vasodilation, augmented sympathetic activity, and baroreflex adaptation (Segar et al., 2006). Importantly, fetal programming of hypertension involves hyper-sympathetic activation of the Ang II type 1 (AT1) receptor activated by endogenous Ang II (Vieira-Rocha et al., 2019). These and other observations support the concept that activation of the renin–angiotensin system plays a role in vascular dysfunction in the adult offspring exposed to glucocorticoids during fetal life (Hsu and Tain, 2021).

Not only intra-uterine, but also early life stress may result in vascular programming, affecting the RAAS system. Adult offspring from mothers submitted to litter separation are more susceptible to hypertension in response to chronic administration of Ang II, as well as to vascular inflammation (Loria et al., 2010) and vascular constriction to Ang II (Loria et al., 2011). Conversely, some protective effects may also be initiated by maternal separation. For example, the capacity of the perivascular adipose tissue to modulate vasoconstriction and endothelial dysfunction increased (Loria et al., 2018). Yet, brief periods of daily maternal separation lead to impaired resistance artery due augmented vascular contractility-response and higher blood pressure in adult rats (Reho and Fisher, 2015). Even the pulmonary vasculature may be affected by stressor conditions, including cross-fostering in early natal period, through renin–angiotensin system modulation (Shifrin et al., 2015).

Renin–angiotensin–aldosterone system may also be a target of fetal programming. The imbalance between the ratio of plasma Ang II to Angiotensin 1–7 [Ang-(1–7)], along with a gradual reduction of Ang-(1–7) were observed in adult offspring from sheep treated with betamethasone during pregnancy, and these alterations occurred prior to blood pressure increases (Shaltout et al., 2012). Yet, antenatal exposure to betamethasone caused a persistent reduction in serum and kidney angiotensin-converting enzyme (ACE)-2 activity, favoring higher blood pressure levels and decreased Ang-(1–7) levels (Shaltout et al., 2009). The impact of fetal programming on the Ang-(1–7) axis was recently reviewed (South et al., 2019).

Dexamethasone administration in fetal life resulted in a compensatory response of endothelium-dependent relaxation, as well as increased contractile response to endothelin-1 (ET-1) (Molnar et al., 2003). Adult offspring from betamethasone-exposed mothers displayed augmented blood pressure, and ET-1 increased blood pressure and vascular resistance *via* ET(A) receptor (Lee et al., 2013). Yet, impaired ET(B) receptor activity favored augmented ET-1 contractility in vascular smooth muscle cells, along with a decrease in ET-1-induced endothelium-dependent relaxation (Pulgar and Figueroa, 2006).

### Epigenetic Events and Vascular Prenatal Programming

Epigenetic events connect the intrinsic genetic background with the extrinsic environment, resulting in the attachment of chemical groups in the DNA, modulating gene expression. The most frequent and described DNA modifications include DNA methylation or hydroxymethylation, histone modifications, and chromatin packaging in addition to the occurrence of microRNAs (miRNAs) and long non-coding RNAs (lncRNAs) (Rane et al., 2009; Deng et al., 2015; Shi et al., 2015).

A growing body of evidence supports the idea that glucocorticoid-related genes involved in augmented cardiovascular risk factors in adult life are targeted by DNA methylation, resulting from early life stressful environment (Friso et al., 2008; Reynolds et al., 2013; Tobi et al., 2018). Another major issue is how the placenta may be affected by epigenetic modifications. In this regard, microRNA interference, DNA methylation, as well as histone methylation suppress placental 11β-HSD2 and GR genes, favoring augmented glucocorticoid levels (Togher et al., 2014).

Considering that undernutrition imposes IUGR and a hypoxic environment to the fetus, the absence of oxygen may regulate a variety of oxygen-regulated genes, *via* HIFs (Adams et al., 2009). miRNAs related to renin–angiotensin system components are affected by maternal hypoxia, and this genetic reprogramming may be involved in the development of pulmonary hypertension in adult offspring (Goyal et al., 2011).

Environmental factors, including ethanol exposure during intrauterine life also result in cardiovascular reprogramming. Ethanol exposure in uterine life reduces 11β-HSD2 expression in the rat placenta and exposes the fetus to glucocorticoids overproduction, by a mechanism that increases permeability of the placental barrier (Zhang et al., 2014). Offspring displayed elevated blood pressure in adulthood and endothelium-dependent and -independent vasodilation was impaired in rats (Turcotte et al., 2002). Morphologically, the vasculature was also modified, and offspring from mothers exposed to ethanol during pregnancy displayed augmented arterial stiffness (Parkington et al., 2014).

Epigenetic alterations, such as DNA methylation and histone modification may be observed upon ethanol consumption (Kleiber et al., 2014; Chater-Diehl et al., 2017). In this regard, long-term DNA methylation was investigated in children displaying neurological and multiple tissue injuries related to prenatal ethanol exposure, and a singular profile of DNA methylation was observed (Laufer et al., 2015). Experiments using the Agouti viable yellow mouse model showed that intrauterine exposure to ethanol generated DNA methylation in the adolescent offspring, as indicated by coat color change (Kaminen-Ahola et al., 2010).

Smoking during pregnancy represents another major concern of how environmental exposure may affect vascular programming. Although most of the data supporting deleterious effects of smoking comes from cigarette smoking, electronic cigarettes may also be important players on fetal programming (Holbrook, 2016). Exposure to nicotine during intrauterine life resulted in DNA methylation of miR-181 contributing to the cardiac ischemia sensitive phenotype in adult rats, favoring autophagy signaling pathways (Jian et al., 2020). AT1 and AT2 receptors gene expression in adult rat offspring from nicotine-exposed mothers is modulated by DNA methylation, eliciting high blood pressure and augmented vascular contractility in adulthood (Xiao et al., 2014). Impaired vascular reactivity due to antenatal exposure may be due to excessive ROS production, *via* Nox-2 dependent mechanisms (Xiao et al., 2011). Translational data showed that preschool children exposed to tobacco during pregnancy display normal blood pressure, but, surprisingly, their systolic blood pressure is increased (4.2 mmHg), compared to that in children with similar age/height that were not exposed to tobacco (Nordenstam et al., 2019).

Recently, particulate matter was also recognized as a possible environmental player in intrauterine stress and programming. On top of the gestational impact that ultrafine particles exposure generates, it also favors programming *via* DNA hypomethylation, eliciting fetal activation of promoters of RAAS, including AT1 receptor and ACE, contributing to increased blood pressure in adult mice offspring. Placental levels of 11β-HSD2 were reduced, whereas maternal and fetal cortisol levels were increased, in the ultrafine particles-exposed group (Morales-Rubio et al., 2019).

In the future, the use of epigenetic markers may be very useful to select the population who is under high risk for cardiovascular events due to intrauterine maternal stress, using biomarker-guidance interventions as a strategy to closely follow these higher risk patients. Remarkably, placental epigenetic biomarkers may also be used to map populations that might be in high cardiovascular risk in adult life.

## Maternal Stress and Fetal Programming of Immune Response

Multiple studies suggest that chronic maternal stress plays have deleterious effects on fetal and neonatal immune functions. Repeated maternal stress significantly alters offspring leukocyte function (Coe et al., 2002), affects placental transfer of maternal antibodies in a sex-dependent manner (Coe and Crispen, 2000), and increases susceptibility to infection (Kay et al., 1998). Although some elements have already been described, it is difficult to define the profile of the programmed immune response due to the different experimental models and the different results described. This chapter highlights the main mechanisms by which maternal stress programs the immune response in the offspring.

When discussing changes in the immune response due to programming originated from maternal stress in the prenatal period, it is essential to consider the windows of vulnerability. These are moments during cell development, where there is a continuous change in gene expression and in the profile of molecules that result in specific time frames in which some cellular elements become more sensitive than others (Veru et al., 2014). Another important observation is that the development and maturation of immune system components occur at different times depending on the studied species. In general, in animals that give birth to preterm offspring (sheep, pigs, guinea pigs, and primates) the development of the immune system occurs predominantly *in utero* (Holladay, 1999; Holladay and Smialowicz, 2000; Merlot et al., 2008). By contrast, in species that give birth to non-preterm offspring (rats, rabbits, and mice), a large proportion of the development occurs during late gestation and in the postnatal period (Holladay, 1999; Holladay and Smialowicz, 2000). For example, the capacity of T cells from 3-day-old monkeys to respond to non-self antigens in mixed lymphocyte cultures is decreased in babies whose mothers were stressed during mid-late pregnancy, and increased in babies whose mothers were exposed to the same stressor during early pregnancy. This reveals that the direction of the alteration depends on the time of exposure to the stressor during pregnancy.

Another factor to consider in the immune response programmed by maternal stress is the type and duration of the insult. A metanalysis of over 300 studies found that different types of stress impact different aspects of immune function. Acute laboratory stressors upregulate innate immunity and downregulate adaptive immunity. Brief exposure to naturalistic stressors, such as academic examinations, shifts function away from cellular immunity (T-helper type 1; Th1) and toward humoral immunity (Th2); and chronic stressors, which are pervasive and insistent, suppress both innate and adaptive immunity (Segerstrom and Miller, 2004).

Epigenetics mechanisms, such as DNA methylation, can modify the immune response across the lifespan in response to maternal stress during pregnancy (Al-Hussainy and Mohammed, 2021). T cell differentiation is controlled by epigenetic mechanisms that regulate Th1 and Th2 identity. Prenatal maternal psychological stress can affect the epigenetic profile of human T cells in a way that affects cytokine production (Al-Hussainy and Mohammed, 2021). Cao-Lei et al. (2014) examined the DNA methylation patterns in T cells of offspring of women exposed to the 1998 Quebec ice storm and found that the genes most commonly methylated are involved in immune system pathways (Cao-Lei et al., 2014). Studies of rhesus macaques revealed differentially methylated regions in both T cells and prefrontal cortex in monkeys who were separated from their mothers after birth (Provencal et al., 2012).

Most studies on immune response dysregulation by maternal stress report increased risk of asthma and allergic diseases in offspring (Douros et al., 2017). A study performed in Northern Italy, in a population of 3,854 children, found that children of mothers who had experienced stressful life events during pregnancy, exhibit moderately increased risk of having wheezing, asthma, eczema and allergic rhinitis during their childhood (de Marco et al., 2012). In fact, maternal prenatal stress can lead to a disequilibrium of Th1/Th2 ratio, in favor of Th2 response, increasing IL-4 and IL-5 in the offspring. and favoring the development of atopic diseases (Pincus-Knackstedt et al., 2006; Veru et al., 2014; Douros et al., 2017).

However, a study performed by Robinson et al. (2021) examined the relationship between the nature and timing of maternal stress in pregnancy and hospitalization due to infections in the offspring. They included 2,141 offspring in the study and the mothers were asked at 18 and 36 weeks gestation about psychological stress events, such as death of a close friend or relative, separation, marital problems, job loss, money problems, and others. The authors found an association between the timing and the type of stress with infection-related hospitalizations. Surprisingly, hospitalizations were observed only in male offspring, showing a sex-specific risk of severe infection in offspring by exposure to maternal prenatal stress. A potential mechanism suggested is that chronic stress may program a pro-inflammatory phenotype in monocytes and macrophages, increasing susceptibility to infections, possibly *via* the hypothalamic–pituitary axis (Marvar et al., 2010).

In addition, study involving 66,203 mother-child pair, emotional stress during pregnancy was associated only with an increased risk of infectious disease (Tegethoff et al., 2011). Elevated stress levels across pregnancy have also been associated with changes in production of pro-inflammatory cytokines in the offspring. Laviola et al. (2004) used a prenatal stress model where rats underwent three 45-min session per day of prenatal restraint stress on gestation days 11–21. Adolescent offspring exhibited higher IL-1β and decreased IL-2 concentrations in spleen and decreased circulating CD4 T cells, CD8 T cells, and CD4/CD8 ratio compared with stress-free animals. Interestingly, an environmental intervention, enriched housing, reversed most immunological alterations, leading to increased IL-2, reduced IL-1β in splenocytes and increased CD4 and CD4/CD8 ratio (Laviola et al., 2004). These findings suggest that the immunological response can be changed by events occurring at multiple stages in development.

Prenatal maternal stress can also impact the neonatal adaptive immunity along generations. Garcia-Flores et al. (2020) used a murine model of prenatal maternal stress across three generations. The authors applied four stress procedures including swimming, restraint, shaking, and white noise for one week in 10 days post-coitum in three generation of dams and evaluated the adaptive immune response of neonates. Results showed a reduction in T cells and B cells, including regulatory CD4 T cells as well as IL-4 and IL-17A producing T cells only in the second generation, but such effects are restored in the third generation. The authors propose that this response is a compensatory mechanism against prenatal maternal stress.

Although this study did not observe an increase in IL-17A in the offspring of stressed pregnant animals, some studies describe the association between IL-17A and chronic stress (Liu et al., 2012; Swardfager et al., 2014; Nadeem et al., 2017). Increased levels of IL-17A measured at 24–28 week gestation were associated with lower birthweight, lower emotional intelligence, lower antenatal maternal attachment, higher prenatal distress, and higher number of life events in pregnant women (Moore et al., 2019).

The dysregulation of the autonomic nervous system is a hallmark of many psychological disorders and elevated levels of norepinephrine directly lead to increased production of IL-17A from T cells (Case et al., 2016). In fact, the central nervous system plays an orchestrated role in inflammation, inducing a direct impact on inflammatory cytokines and immune cells. The central inhibition of the sympathetic nervous system (SNS) decreased peripheral TNF-α serum levels in hypertensive post-menopausal women (Pöyhönen-Alho et al., 2008). Suppression of the adaptive immune system or sympathetic outflow inhibits hypertension (Rodríguez-Iturbe et al., 2002; Lob et al., 2010), and enhances T cell activation (Marvar et al., 2010) in experimental models. Renal sympathetic nerves play an important role in activation of adaptive immunity in hypertension by T cell activation. In fact, renal denervation not only reduces the total number of immune cells in the kidney, but also memory T cells (Xiao et al., 2015). The immune cells present adrenergic receptors, which have been implicated in sympathetic response in the inflammatory response. Has been reviewed by Kohm and Sanders (2001) that NE and beta 2-adrenergic receptor contributed to CD4 + T and B lymphocyte regulation.

Another mechanism by which the immune response is altered by fetal programming is oxidative stress. ROS generation is an autolimited mechanism of inflammation. However, prolonged inflammation and disturbance in immune homeostasis can lead to an oxidative stress condition and more severe damage of cellular and tissue structures. Restraint stress at late-stage of pregnancy in rats caused increased intracellular ROS, loss of hippocampal neurons and activation of NF-kB signaling (Zhu et al., 2004; Cai et al., 2007). In the same animal model, oxidative damage in mitochondrial DNA in hippocampal neurons only in females rat offspring was reported (Song et al., 2009). Higher levels of glucocorticoid exposure by 11β-HSD inhibitor, during the last week of murine gestation, increased aortic superoxide anion production (Roghair et al., 2011). Treatment of the pregnant mice with Tempol, an antioxidant, did not correct glucocorticoid-programmed aortic superoxide production, but attenuated conditioned fear and stress reactivity.

Maternal obesity can also affect the inflammatory response in the offspring. Obesity, *per se*, is known to be a chronic inflammatory state and C-reactive protein (CRP) is elevated in non-obese adult offspring of two obese parents (Lieb et al., 2009). In another study, levels of high sensitivity-CRP were higher in 12-year-old children exposed to maternal obesity during pregnancy compared to not exposed (Leibowitz et al., 2012). In a study of 189,783 Swedish children, a higher maternal body mass index (BMI) was associated with a higher risk of asthma (Lowe et al., 2011).

Despite the variation in the immune response programmed by maternal stress *in utero*, the most consistently replicated findings have been observed in cytokine homeostasis, where prenatal maternal stress induces a Th2 cytokine shift (adaptive immunity) and excessive pro-inflammatory cytokine responses (innate immunity).

## Programmed Immune Response Contributing to Vascular Dysfunction

**FIGURE 2:**
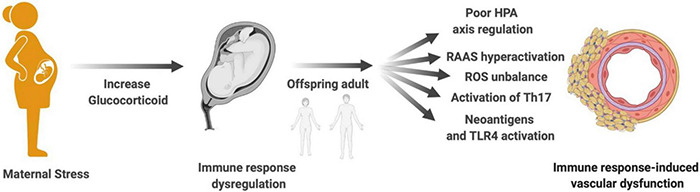
Maternal stress, immune response, and vascular dysfunction. Mechanisms proposed to explain how maternal stress programming-induced immune response dysregulation in the offspring contributes to vascular dysfunction in adult life: poor HPA axis regulation, RAAS hyperactivation, ROS imbalance, activation of Th17, generation of neoantigens, and TLR4 activation. HPA, hypothalamic–pituitary–adrenal; RAAS, renin–angiotensin–aldosterone system; ROS, reactive oxygen species; Th17, T helper lymphocyte 17; TLR4, toll-like receptor 4.

Most cases of arterial hypertension have an idiopathic and multifactorial cause (Whelton et al., 2018). The contribution of immune response activation to increased blood pressure and cardiovascular diseases has already been well described and revised (Bomfim et al., 2017). Thus, the focus of this chapter is to connect the mechanisms of the immune response that contribute to vascular dysfunction and that can be programmed by maternal stress during pregnancy. Here, potential mechanisms whereby programmed immune response induced by maternal stress trigger vascular dysfunction and increase the risk of cardiovascular disease in adulthood will be discussed (Figure 2).

### Poor Hypothalamic–Pituitary–Adrenal Axis Regulation of Inflammatory Response

Glucocorticoids are classically known for their immunosuppressive effect. However, several studies have also shown that chronic treatment with synthetic corticosteroids increases blood pressure (Goodwin and Geller, 2012). The main mechanisms by which corticosteroids increase blood pressure are sodium reabsorption in the proximal tubules of the kidneys, and increased intravascular fluids due to glucocorticoid-induced activation of mineralocorticoid receptors. High doses of dexamethasone reduce 11β-HSD2 expression, increasing susceptibility to hypertension (Bailey, 2017). In addition, glucocorticoid receptors are expressed in vascular smooth muscle cells and endothelium, and their activation changes the vascular behavior, with increased contraction and decreased vascular relaxation. However, little is known about the role of glucocorticoid receptors in these sites (Grossman and Messerli, 2012).

Despite the fact that glucocorticoids have an immunosuppressive function, some studies have shown a pro-inflammatory action of this hormone (Shimba and Ikuta, 2020a,b). One of the mechanisms for the constant activation of the immune system, even at high concentrations of glucocorticoids, is the poor regulation of cytokine-glucocorticoid negative feedback, which influences the relationship between maternal stress during pregnancy and offspring outcomes (Hantsoo et al., 2019). A well-regulated, flexible immune system responds appropriately to glucocorticoid stimulation, and a brief spike in pro-inflammatory cytokines in response to acute stress is considered physiologically appropriate. However, an exaggerated or prolonged immune response is maladaptive, and chronic stress may result in glucocorticoid receptor resistance, the sensitivity of the immune cells to glucocorticoid hormones and inflammatory response (Cohen et al., 2012). Without sufficient glucocorticoid regulation, the duration and/or intensity of the inflammatory response increases. Vascular dysfunction is correlated with elevated serum levels of inflammatory markers and increased immune response (Shao et al., 2020), and glucocorticoid receptor resistance increases the intensity and duration of inflammatory processes, further contributing to vascular dysfunction and heightening risk and progression of cardiovascular disease results in failure to downregulate inflammatory response.

### Activation of T Helper-17 Cells

T helper-17 (Th17) cells, a unique CD4+ T-cell subset, are important in the secretion of IL-17, IL-21, and IL-6. Apart from Th17 cells, selective cell subtypes also produce IL-17, including γδ T cells, NK, and NK-T. IL-17A signaling activates various downstream pathways, which include MAPK and NF-κB to induce mediators with relevance to cardiovascular risk such as CXC chemokines, CXCL1 and CXCL2, involved in the attraction of neutrophils, and inflammatory factors, like IL-6 and granulocyte–macrophage colony-stimulating factor (Tesmer et al., 2008).

In the inflammation-induced stress system, the production of Th1/pro-inflammatory cytokines by Th2 protects the organism from systemic “overshooting” (Elenkov et al., 2005). Although in the Th1/Th2 dichotomy the Th2 response predominates in animals whose mother was submitted to stress, glucocorticoids have also the potential to enhance the development and function of Th17 cells (Shimba and Ikuta, 2020a). Th17 cells are resistant to apoptosis and to the suppression of cytokine production by glucocorticoid treatment (Banuelos et al., 2017). Thus, stress-induced glucocorticoids might trigger inflammation *via* induction of Th17 cells (Shimba and Ikuta, 2020a). Plasma levels of IL-17A are increased in humans with hypertension (Madhur et al., 2010). Mice lacking IL-17A develop blunted hypertension and do not develop endothelial dysfunction in response to Ang II infusion. IL-17A can inhibit endothelial NO production, increase ROS formation, and promote vascular fibrosis contributing to vascular dysfunction (Davis et al., 2021). IL-17A can also cause autoimmunity through an IL-6 positive feedback loop (Ogura et al., 2008). Thus, it is reasonable to hypothesize that Th17 cells are more activated in the offspring of mothers that underwent stressful insults during pregnancy, and that Th17 cells contributes to the onset of vascular dysfunction and cardiovascular disease in adulthood.

### Renin–Angiotensin Aldosterone System Activation

The Ang II axis of the RAAS is recognized as a key pathway in the cardiovascular pathology of fetal programming events. Franco Mdo et al. (2003a) showed that Ang II concentration is increased in mesenteric arteriolar beds from intrauterine undernourished rats. The same experimental model exhibits augmented ACE activity (Langley-Evans et al., 1999). Reduced ACE2 activity and increased Ang II/Ang-(1–7) ratio were found in adult sheep offspring whose mothers were exposed to betamethasone, in the third trimester of pregnancy. Therefore, exposure to glucocorticoids, likewise betamethasone, programs a dysfunctional RAAS (South et al., 2019), implicating in higher long-term risk for hypertension and cardiovascular disease.

Angiotensin II acts mainly through AT1 receptor and activates many mechanisms in the kidney, nervous system, and blood vessels that cause hypertension. Besides its classic role in the regulation of circulatory homeostasis, it is very well described that Ang II is also a powerful pro-inflammatory mediator (Biancardi et al., 2017). Ang II inflammatory effects are mediated by activation of NF-κB, and the production of inflammatory mediators, including interleukin IL-1β, IL-6, and TNF-α (Marchesi et al., 2008). Ang II, *via* AT1 receptor, also stimulates NADPH oxidase (Nox enzymes) and generates ROS in the blood vessels (Schiffrin and Touyz, 2003). In response to Ang II, the expression of several inflammatory chemokines and their receptors are increased in the target organs, including the vasculature, mediating homing of immune cells (Mikolajczyk et al., 2021). Therefore, it is plausible to suggest that the RAAS is an important immumodulator that contributes to vascular dysfunction in offspring due to maternal stress in the prenatal period.

### Reactive Oxygen Species Imbalance

As already discussed, maternal stress *in utero* increases ROS generation and leads to chronic oxidative stress in various organs of the adult offspring. The production of ROS is strongly stimulated during activated immune responses, due to the important microbicidal activity of ROS. However, the exaggerated and chronic ROS production damages the vascular system. There are excellent reviews covering the role of oxidative stress, vascular dysfunction, and hypertension (Higashi et al., 2014; Montezano et al., 2015; Griendling et al., 2021). In general, oxidative stress favors a vasoconstrictor, mitogenic, pro-fibrotic, pro-migratory, and pro-inflammatory phenotype in endothelial cells and vascular smooth muscle cells (Montezano et al., 2015). With this in mind, it is very likely that adults will develop vascular dysfunction triggered by programmed oxidative stress during the pregnancy of stressed mothers.

### Generation of Neoantigens and Toll-Like Receptor-4 Activation

In addition to directly causing vascular dysfunction, oxidative stress, or the excess of ROS can oxidize endogenous molecules and generate damage-associated molecular patterns (DAMPs), such as oxidized low-density lipoprotein (oxLDL) and oxidized phospholipids (oxPL) (Rocha et al., 2016). oxLDL binds to TLR4, which is a key signaling receptor of innate immunity (Miller et al., 2005). TLR4 signaling leads to activation of MAPK and NF-κB, resulting in secretion of pro-inflammatory cytokines such as TNF-α, IL-6, IL-1β, and others (Kawai and Akira, 2006). oxLDL activates TLR4 and induces secretion of chemokine IL-8 and monocyte chemoattractant protein-1 (MCP-1) by human endothelial cells and monocytes (Miller et al., 2005). In addition, oxLDL/LDL-C ratio, an atherogenic index, is increased in IUGR fetuses compared to neonates of healthy mothers with appropriate weight.

Activation of TLR4 is directly involved in vascular inflammation, vascular dysfunction, and hypertension (Bomfim et al., 2012, 2015). Vascular TLR4 expression is increased in cardiovascular disease, including several hypertension (SHR, Ang II infusion, DOCA-Salt), atherosclerosis, and other animal models (Roshan et al., 2016; Biancardi et al., 2017). Treatment with a neutralizing anti-TLR4 antibody decreases blood pressure and vascular inflammation in hypertensive rats (Bomfim et al., 2012, 2015). TLR4 overexpression aggravates vascular smooth muscle cells proliferation and vascular remodeling in the pathogenesis of hypertension (Qi et al., 2021).

In view of all these information, we suggest an alternative mechanism involved in vascular dysfunction programmed by immune response in offspring submitted to stress *in utero*: the generation of neoantigens by oxidative stress and activation of TLR4.

## Conclusion

In conclusion, this review proposes a connection between maternal stress, immune response and vascular dysfunction. During pregnancy, stressors occurring in different gestational periods activate immune responses in the offspring, favoring a poor HPA axis regulation of inflammation, RAAS activation, unbalanced ROS, and generation of new antigens and activation of TLR4. All these mechanisms are directly involved in vascular dysfunction and cardiovascular disease development.

Thus, a careful attention/assistance to women during pregnancy and the nursing period is essential to program a healthy cardiometabolic phenotype in the offspring. In fact, by giving special attention to women living in socially unassisted communities is an urgent necessity around the world. Strategies to implant social policies that can improve the nutritional status, educational strategies to avoid the use of licit drugs like alcohol and tobacco, as well as especial attention to the psychiatric and behavioral complications in this stage can avoid maternal stress and the long-term impact of negative events in the offspring adult life. Avoiding maternal stress is an important step to prevent the onset of cardiovascular diseases in the offspring and their long-term consequences.

## Author Contributions

GB, TC, FG, and JD conceptualized the idea for the manuscript. GB, TC, FG, JD, and VL performed the literature search and drafted different sections of the manuscript. RT, GB, and TC revised and edited the final version of the manuscript. TC drafted the figures. All authors contributed to the article and approved the submitted version.

## Conflict of Interest

The authors declare that the research was conducted in the absence of any commercial or financial relationships that could be construed as a potential conflict of interest.

## Publisher’s Note

All claims expressed in this article are solely those of the authors and do not necessarily represent those of their affiliated organizations, or those of the publisher, the editors and the reviewers. Any product that may be evaluated in this article, or claim that may be made by its manufacturer, is not guaranteed or endorsed by the publisher.

## Abbreviations

ACE: angiotensin-converting enzyme
Ang II: angiotensin II
AP-1: activator protein-1
AT1: Angiotensin II receptor type 1
Ang-(1–7): angiotensin (1–7)
BH4: tetrahydrobiopterin
CRP: C-reactive protein
CXC: chemokines
CXCL1: chemokine (C-X-C motif) ligand 1
CXCL2: chemokine (C-X-C motif) ligand 2
DAMPs: damage-associated molecular patterns
DOHaD: developmental origins of health and diseases paradigm
ET-1: endothelin-1
ET(A): endothelin-1 receptor type A
ET(B): endothelin-2 receptor type B
eNOS: endothelial nitric oxide synthase
GILZ: glucocorticoid-induced leucine zipper
GR: glucocorticoid receptor
HIF-1: hypoxia-inducible factor 1
HPA: hypothalamic–pituitary–adrenal axis
ICAM-1: intercellular adhesion molecule-1
IUGR: intrauterine growth restriction
IL-1 β: interleukin-1 β
IL-6: interleukin-6
IL-8: interleukin-8
IL-15: interleukin-15
IL-17A: interleukin-17A
IRF: type I interferon (IFN)-regulatory factor
lncRNAs: long non-coding RNAs
MAPK: mitogen-activated protein kinase
MCP-1: monocyte chemoattractant protein-1
miRNAs: microRNAs
MKP-1: MAPK phosphatase-1 or dual specificity phosphatase-1 (DUSP-1)
NADPH oxidase: nicotinamide adenine dinucleotide phosphate hydrogen oxidase
NK: natural killer cells
NF- κ B: nuclear factor kappa B
NO: nitric oxide
⋅ O_2_^–^: superoxide anion
oxLDL: oxidized low-density lipoprotein
oxPL: oxidized phospholipids
RAAS: renin–angiotensin–aldosterone system
ROS: reactive oxygen species
Th1: T-helper type 1 cell
Th2: T-helper type 2 cell
Th17: T helper-17 cell
TLR: toll-like receptors
TNF- α: tumor necrosis factor alpha
VEGF: vascular endothelial growth factor
11 β-HSD 2: 11 beta-hydroxysteroid dehydrogenase type 2 enzyme.
